# Roost Disturbance and Predation: Agama Lizard (*Agama* sp.) Preying on Slit‐Faced Bats (*Nycteris* sp.) in Zakouma National Park, Chad

**DOI:** 10.1002/ece3.72246

**Published:** 2025-10-15

**Authors:** Elsa M. S. Bussière, Cecilia Montauban, Cyril Pélissier, Cristian Pizzigalli

**Affiliations:** ^1^ FitzPatrick Institute of African Ornithology University of Cape Town Rondebosch South Africa; ^2^ Department of Life Sciences Imperial College London Ascot UK; ^3^ Department of Life Sciences Natural History Museum London London UK; ^4^ Greater Zakouma Ecosystem African Parks Network Zakouma Chad; ^5^ CIBIO, Centro de Investigação em Biodiversidade e Recursos Genéticos Universidade do Porto Vairão Portugal; ^6^ Program in Genomics, Biodiversity and Land Planning, CIBIO BIOPOLIS Vairão Portugal; ^7^ Departamento de Biologia da Faculdade de Ciências Universidade do Porto Porto Portugal; ^8^ Biosciences, Faculty of Health and Life Sciences University of Exeter Exeter UK

**Keywords:** predator–prey interactions, Sahel, Sudanian savannah, synanthropy, trophic networks

## Abstract

Predation plays a key role in shaping ecological interactions, particularly in environments where seasonal dynamics drive adaptive behaviors. In semi‐arid Sudano‐Sahelian ecosystems, where resource availability fluctuates significantly, predator–prey interactions are influenced by both natural and anthropogenic pressures. This study documented the first recorded instance of an agama lizard (*Agama* sp.) preying on a slit‐faced bat (*Nycteris* sp.) in Zakouma National Park, Chad. The event occurred in March 2024, during the dry season, after a colony of *Nycteris* bats was displaced from a hut roost by olive baboons ( *Papio anubis* ), forcing them to relocate to a more exposed building. The bats' increased visibility and reduced cover likely increased their vulnerability to predation by the agama, a diurnal opportunistic predator commonly found around human settlements. Over four recorded consecutive predation attempts, the agama displayed behaviors including biting and dragging bats, until it finally captured one. All three species involved (*Agama* sp., *Nycteris* sp. and *P. anubis* ) are synanthropic, and their shared use of human‐modified environments may have facilitated this interaction. This observation underscore the ecological flexibility of agama lizards and highlight how synanthropy can drive novel predator–prey dynamics. Our findings contribute to a growing body of evidence of reptilian predation on bats and emphasize the importance of understanding food web dynamics in increasingly altered African savannah ecosystems.

## Graphical abstract

We report the first documented case of an agama lizard (*Agama* sp.) preying on slit‐faced bats (*Nycteris* sp.). This rare observation occurred during the dry season in Zakouma National Park, Chad, and may have been triggered by the displacement of bats from their roost by olive baboons ( *Papio anubis* ). This interaction highlights how synanthropy and seasonal dynamics can drive novel predator–prey relationships in human‐modified Sudano‐Sahelian ecosystems. It also raises questions about whether bats serve as opportunistic prey or a regular component of Agama diets in these environments, underscoring the need for further research to better understand trophic dynamics in these understudied systems.

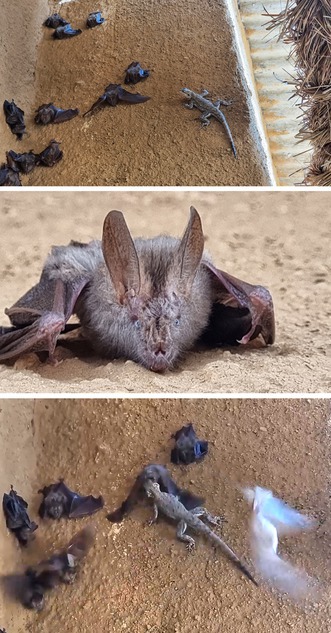

## 1 Introduction

Predation is a fundamental ecological process that shapes community structure, regulates prey populations, and drives evolutionary adaptations (Abrams 2000; Rikvold and Sevim 2007; Chen et al. 2011; Schmitz 2017; Colombo et al. 2019; Araujo et al. 2020). In dynamic environments such as semi‐arid Sudano‐Sahelian savannah ecosystems, where there are extreme oscillations of temperature and resource availability, predator–prey interactions are mediated by both natural cycles and anthropogenic influences (Letnic et al. 2011; Zomer et al. 2022). These landscapes experience short, intense rainy seasons followed by long dry periods, creating challenging conditions for survival. Many species in such systems respond through behavioral flexibility or seasonal movement. For example, large herbivores such as elephants and antelopes migrate seasonally to track vegetation and water (Birkett et al. 2012; Wato et al. 2018), or might adjust their habitat use seasonally, like some giraffe populations that shift between wooded and open habitats in response to changing resource availability (Clark et al. 2023). Other species adapt in situ by modifying their activity or energy expenditure. For instance, many amphibians and reptiles exhibit hypometabolic strategies such as aestivation/siccatation or reduced foraging to cope with seasonal resource scarcity (Gil et al. 1993; Christian et al. 2007; Secor 2005; Yoshida and Kaito 2020; Jiang et al. 2023).

This ecological flexibility extends to predator–prey interactions, which are not only shaped by seasonal fluctuations but also increasingly influenced by human‐altered landscapes (Pafilis and Valakos 2003; El‐Sabaawi 2018; Fleming and Bateman 2018). In semi‐arid environments, predators often adapt their foraging strategies to exploit temporal peaks in prey availability; for example, raptors modify their hunting behavior to take advantage of increased prey activity after rainfall events (Ferreira and Faria 2021). Such opportunistic responses highlight the dynamic nature of predation. Moreover, the expansion of synanthropic environments—those shaped by human activity—further complicates these dynamics, in part by bringing together species that may not normally co‐occur (Johnson and Munshi‐South 2017; Alberti et al. 2020). In these human‐modified settings, the potential for novel or opportunistic predator–prey interactions increases, as predators exploit new opportunities presented by the altered landscape (Fleming and Bateman 2018). Documenting and understanding these interactions is essential for guiding future research, particularly in ecosystems undergoing rapid change due to climatic extremes and human influence.

While substantial research has focused on mammals and birds as predators, far less is known about the predatory behavior of reptiles, particularly in semi‐arid regions. Lizards are key components of arid and semi‐arid ecosystems, functioning as seed dispersers (Valido and Olesen 2019), ecosystem engineers (de Miranda 2017), and mesopredators that shape vertebrate and invertebrate community dynamics (Panov and Zykova 2016). Agamid lizards are among the most species‐rich lizard families (Uetz and Hošek 2025), successfully adapted to a wide range of habitats (Greer 1989; Panov and Zykova 2016; Tan et al. 2020), including human‐altered environments, where they have been shown to exhibit synurbic behaviors and adjust their foraging strategies (Whiting et al. 1999; Singh et al. 2021). Agamids are generally insectivorous, with ants being their most significant food source (Tan et al. 2020), but they also display opportunistic feeding behavior, particularly in periods of resource scarcity (Chapman and Chapman 1964; Cloudsley‐Thompson 1981; Ofori et al. 2018). Moreover, agamids rarely forage beyond their home ranges (Whiting et al. 1999), indicating that their diet may be influenced by the food availability in the microhabitats they occupy. Agama lizards have been reported preying on small vertebrates, including their own young, other lizards, snakes, birds, and mammals (Harris 1964; Cloudsley‐Thompson 1981; Roumelioti et al. 2025). However, no prior published record exists of predation on bats by agamas.

Bats (Mammalia: Chiroptera), despite being highly mobile and capable of flight, are not immune to predation. They can form some of the largest mammalian aggregations and represent a substantial potential food source for a wide range of predators (Kunz 2003; Jones et al. 2009; Kasso and Balakrishnan 2013). Bats' nocturnality is often considered an evolutionary strategy to minimize predation risk, particularly from diurnal predators (Speakman 1991; Rydell et al. 1996; Kunz and Fenton 2003; Mikula et al. 2016). Their susceptibility to predation is closely linked to their temporal activity patterns, roosting habits, and the presence of predators with diverse hunting strategies (Lima and Dill 1990; Arndt et al. 2018). Bats are vulnerable to predation while roosting, particularly in exposed locations (Mikula 2015; Esbérard and Vrcibradic 2007). Traditionally considered to have few natural predators (Speakman 1995; Rydell et al. 1996), bats are now recognized as prey for a surprisingly wide range of animals, including birds (Speakman 1991; Mikula et al. 2016), mammals (Ancillotto et al. 2013; Welch and Leppanen 2017; Mori et al. 2019; Oedin et al. 2021; Borkin et al. 2023; Labadie et al. 2024), arthropods (Molinari et al. 2005; Nyffeler and Knörnschild 2013; Noronha et al. 2015; Ruiz‐Villar et al. 2024), amphibians (Mikula 2015), fish (Mikula 2015), and reptiles (Esbérard and Vrcibradic 2007; Shirley et al. 2016; Barti et al. 2019). However, the role of reptiles in bat predation is still poorly understood, likely due to the rarity of observed events and the challenges of documenting such interactions in the field.

Here, we report the first documented instance of a lizard of the genus *Agama* (possibly *A. boueti* Chabanaud, 1917) preying on a bat of the genus Nycteris, G. Cuvier & E. Geoffroy, 1795. This observation occurred following roost disturbance by a third species, which forced bats to relocate to a more exposed site. This interaction contributes to the growing body of literature on bat predation, expands our understanding of agamid dietary flexibility, and underscores the role of synanthropy and habitat disturbance in shaping novel predator–prey relationships in semi‐arid ecosystems.

## 2 Methods and Results

Zakouma National Park, established in 1963, is located in the Sudano‐Sahelian region of Chad, in a semi‐arid habitat characterized by extreme seasonal fluctuations. The park experiences a stark contrast between its dry and wet seasons. From October to April, the region endures a prolonged dry season. At the end of the dry season, from March to April, the environment becomes exceptionally dry, with water scarcity posing a significant challenge for wildlife. However, the situation changes dramatically from May to September, when the area receives heavy rainfall, transforming the landscape. The seasonal rains and rising water levels in catchment areas lead to substantial flooding, reshaping the environment and creating temporary wetlands. This annual cycle of extreme wet and dry conditions plays a pivotal role in shaping the dynamics of the ecosystem (Mahmood et al. 2020; Njouenwet et al. 2022).

**FIGURE 1:**
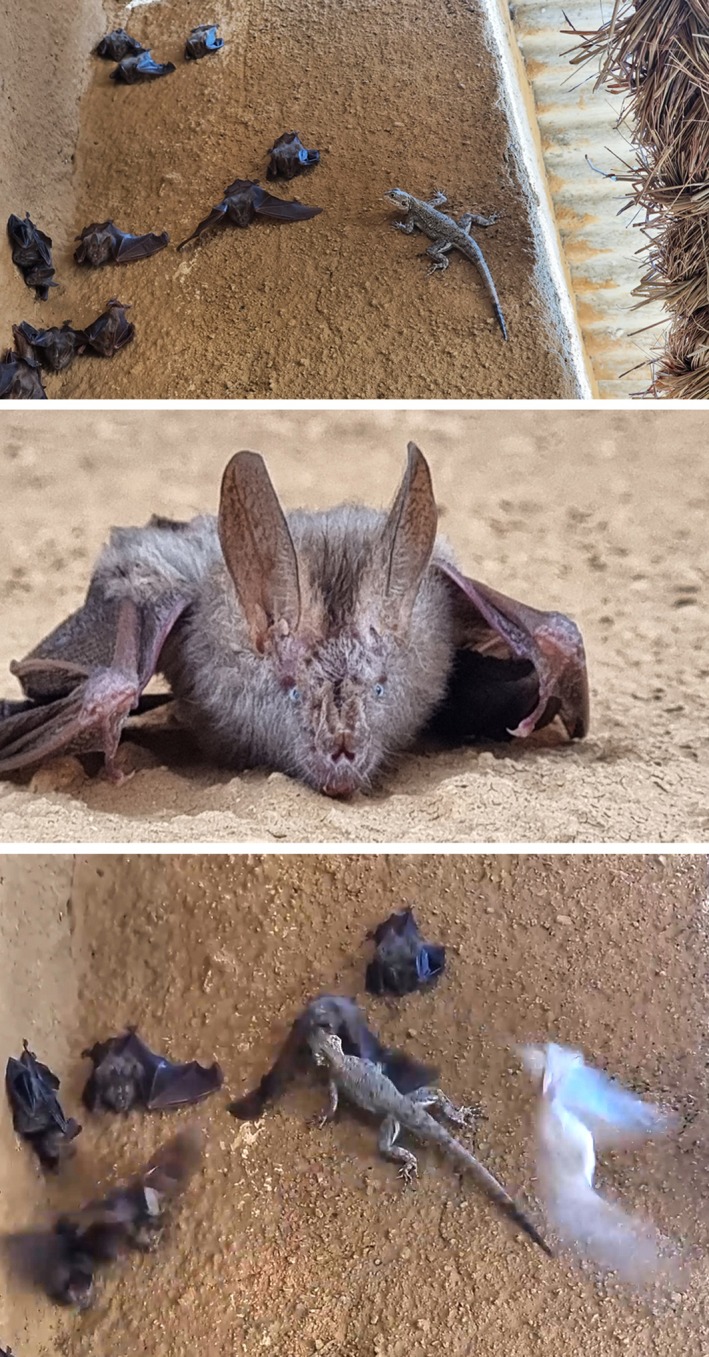
Photographs documenting the predation event at Zakouma National Park, Chad, on March 4, 2024. From top to bottom: (1) the agama lizard (*Agama sp.*) approaching a group of approximately 15 slit‐faced bats (*Nycteris sp.*) roosting exposed on a wall following a recent disturbance; (2) a close‐up view of the slit‐faced bats; and (3) the moment of predation, showing the agama biting one of the bats.

The observed predation took place at the park's headquarters (GPS coordinates: 10.88857° N, 19.81927° E) on March 4, 2024, during the dry season. The headquarters is a fenced camp located within Zakouma National Park, with housing structures made of parpins, covered with a mixture of cement and soil plaster. At 14:10 local time, an agama lizard (*Agama* sp.) approached a group of approximately 15 slit‐faced bats (*Nycteris* sp.) roosting on the exterior southwest‐facing wall of a house under the cover of a thatch roof at a height of about two meters (Figure 1). Based on the location, the slit‐faced bat species is likely *Nycteris thebaica* or *Nycteris hispida* (Monadjem et al. 2024), but identification would require capturing and handling the bat to take measurements and examine its morphology. This was not done to avoid disturbing the colony further. The house is right next to an artificial perennial water source, a game‐viewing waterhole associated with the camp. The event was documented through photo and video capture using a Samsung Galaxy S21 Ultra 5G Android smartphone (Samsung Electronics, South Korea).

The bats had recently relocated to this new roosting site after their original roost—a small thatched hut located 10 m away and used as a laundry room—was damaged by a troop of olive baboons, *Papio anubis* , the previous day. The baboons entered the hut and destroyed the roof, forcing the bats to relocate to an alternative roosting site.

The agama descended from the top of the wall beneath the metal roof, approaching the bat colony from above. As it neared the roosting bats, it adjusted its posture to face them and advanced in short, rapid bursts, interspersed with pauses of varying duration (Video 1). During these pauses, it elevated the anterior part of its body and cocked its head. The bats initially remained unresponsive. There were four predation attempts by the same agama on the roosting bats (Video 1). On the first attempt, the agama bit a bat's head but immediately released its grip and moved away towards the top of the wall when the bat took flight. It then immediately descended again, possibly in search of another opportunity. In the second and third attempts, the agama lunged forward, attempting to grasp a bat by the dorsal side, and successfully dragged the bats to the top of the roof. Although there was no clear visual on what happened next, the fact that the agama reappeared almost immediately indicates it is likely that the bats managed to escape the grip of the lizard. On the fourth attempt, the agama firmly seized the tip of a bat's left wing and climbed the wall while carrying it, managing to hold onto the bat despite the slit‐faced bat's rapid movements attempting to escape (Video 1).

**VIDEO 1:** *Agama* sp. preying on *Nycteris* sp. in Zakouma National Park, Chad (March 4, 2024). Over four consecutive predation attempts recorded on video, the agama exhibited behaviors such as biting and dragging individual bats, ultimately managing to seize one; however, the final outcome of the predation event is unknown. Video content can be viewed at https://onlinelibrary.wiley.com/doi/10.1002/ece3.72246.

The final outcome of this interaction was not observed, as both the agama and the bat disappeared onto the roof. However, unlike its previous attempts, the agama did not return. Following each predation attempt, the remaining bats exhibited erratic flight behavior around the agama before settling back to the same roosting location.

## 3 Discussion

This observation represents the first documented case of a lizard of the genus *Agama* preying on bats, highlighting the species' opportunistic foraging behavior and documenting a previously unknown predator of bats. While primarily insectivorous, this predatory behavior highlights the great dietary flexibility of Agama lizards, with small vertebrates playing a potential role in supplementing their diet. The interaction also underscores the role of roost disturbance and destruction in exposing bats to novel predation risks.

Roosting bats are particularly vulnerable to predation, as their defensive options are limited while at rest (Boinski and Timm 1985; Sparks et al. 2003; Veilleux et al. 2003; Estók et al. 2009). The original roosting site—a small thatched hut—likely provided some level of concealment, but its destruction by olive baboons forced the bats to relocate to a more exposed setting on a house wall. This shift may have increased their susceptibility to diurnal predators such as agama lizards. Notably, the Nycteris bats were resting in an unusual roosting position at the time of observation—they were roosting flat against the wall with wings spread out, rather than free‐hanging from the roof as is more typical for the genus. This atypical positioning may have been a stress response to the recent disturbance and predation threat, or could reflect a thermoregulatory strategy to dissipate heat in the exposed hot environment (Stones and Wiebers 1965).

Slit‐faced bats commonly roost in human‐made structures like buildings and culverts, but also utilize a diverse array of natural roosts, including hollow trees, caves, and burrows (Monadjem 2006; Monadjem et al. 2009, 2020; Hall 2023; MacDonald 2024). Many questions remain about bat roosting ecology, including understanding the factors associated with roost selection, and how that is changing in increasingly anthropogenic environments (Voigt and Kingston 2016). The synanthropic nature of all three species involved—the bat, the agama, and the baboon—further highlights how human‐modified environments can alter predator–prey dynamics (Dorresteijn et al. 2015; Wilson et al. 2020; Van Scoyoc et al. 2023). The use of human‐made structures by bats increases the likelihood of disturbance by humans and other synurbic species, which may, in turn, elevate predation risks during daylight hours when bats would generally be concealed.

Predators can exert both direct and indirect influences on prey populations, shaping not only their abundance but also their behavior and habitat use. Bats, with their long lifespans and low reproductive rates (Kunz and Fenton 2003), are particularly sensitive to increased mortality risks. Unlike many other animals that navigate a constant trade‐off between foraging efficiency and predation risk, bats typically exploit aerial habitats with relatively few nocturnal predators. This suggests that they experience lower levels of anti‐predator pressures compared to many other vertebrates. However, events such as roost disturbance and subsequent exposure to unexpected diurnal predators may introduce new selective pressures that could influence their behavior and roosting ecology.

Notably, our video recordings reveal a progression in the agama's predatory strategy over the four documented attempts. Initially, the agama released the bat immediately after catching it but progressively increased its effort in handling the prey. In subsequent attempts, it began to grab and carry the bat towards the roof, returning quickly after each escape. By the fourth attempt, the agama successfully grasped the bat's wing, likely making it more challenging for the bat to flap and escape compared to previous attempts. This additional difficulty seemed to enhance the agama's ability to carry the bat upward, demonstrating a more determined effort to secure its prey. Animals enhance their survival and reproduction by making adaptive decisions that integrate ecological information to optimize their behavior and fitness. We observed lip‐licking behavior, which may indicate the agama was gathering chemical information via the Jacobson's organ to assess prey suitability, a behavior that has been documented in other lizards (Broman 1920; Kahmann 1932; Wilde 1938; Kubie et al. 1978; Schwenk 1995). This behavior suggests that the lizard was initially uncertain, reinforcing the unusual nature of this predation event. However, studies have shown that lizards tend to become more proactive in their foraging activity when environmental conditions are harsher (Drakeley et al. 2015), which could be the case during this period at the peak of the dry season, when food availability might have been scarce.

This case highlights the need to expand both the geographic and taxonomic scope of research on lizard predatory behavior and the diversity of bat predators in different environments. While some bat predators are relatively well documented, reptilian predation on bats remains poorly understood, likely due to limited direct observations and unpublished accounts. The darkness and remoteness of where these interactions occur also play a role in the scarcity of records. Expanding our knowledge of reptile‐bat predator–prey interactions across diverse ecosystems is essential for refining our understanding of ecosystem interactions, prey vulnerability, and the cascading effects of predation within food webs. Although the final outcome of this predation attempt was not observed, this finding underscores the heightened risk bats face following habitat disturbances, which can expose them to novel threats. It also calls for further investigation into the dietary ecology of agama lizards and the broader implications of habitat disruption on bat survival. As human activity continues to reshape landscapes, understanding how environmental disturbances influence predator–prey dynamics and other biotic interactions will be critical for assessing threats to wildlife populations and revealing the complex dependencies that underpin ecological balance and resilience.

## Author Contributions

**Elsa M. S. Bussière:** data curation (lead), writing – original draft (equal), writing – review and editing (lead). **Cecilia Montauban:** writing – original draft (equal), writing – review and editing (supporting). **Cyril Pélissier:** data curation (supporting), writing – review and editing (supporting). **Cristian Pizzigalli:** writing – original draft (equal), writing – review and editing (supporting).

## Conflicts of Interest

The authors declare no conflicts of interest.

## Data Availability

All relevant data, including selected photo and video materials, is included in the note. Edited segments were removed for clarity and conciseness without withholding substantive content. For additional information or unedited materials, contact Elsa Bussière at elsabussiere@gmail.com.
